# Morphological Parameters of 29994 sperm in a fertile male population—based on Papanicolaou staining and SSA-II Plus

**DOI:** 10.3389/fendo.2025.1546290

**Published:** 2025-03-07

**Authors:** Longlong Fu, Fang Zhou, Guoping Chen, Renpei Yuan, Wenjie Li, Shi Qiu, Liang Tang, Wenshu Liu, Yiqun Gu, Wenhong Lu

**Affiliations:** ^1^ Reproductive Health Research Centre, National Research Institute for Family Planning, Beijing, China; ^2^ Hunan Suiplus Medical Technology Co., Ltd, Changsha, China; ^3^ Department of Urology, The Third Hospital of Beijing University, Beijing, China; ^4^ Reproductive Medicine Centre, Jiangxi Maternal and Child Health Hospital, Nanchang, China; ^5^ School of Ethnology and Sociology, Minzu University of China, Beijing, China

**Keywords:** sperm morphology, computer-assisted sperm analysis (CASA), Papanicolaou staining, male infertility, fertility mens

## Abstract

**Objective:**

This study aims to provide reference data for sperm morphology in a healthy, fertile male population providing a foundation for future studies on male infertility assessment and sperm selection in assisted reproductive technologies.

**Methods:**

The study included 21 healthy male participants, all of whom had partners who conceived within the past 12 months. Sperm samples were collected according to WHO guidelines and stained using the Papanicolaou method. Sperm morphology parameters, including head length, width, area, perimeter, ellipticity, and acrosome area, were measured using the Suiplus SSA-II Computer-Assisted Sperm Analysis (CASA) system. Statistical comparisons were made between CASA and traditional manual methods.

**Results:**

The percentage of sperm with normal head morphology was 9.98%. Detailed sperm head measurements, including length, width, and area, were provided as reference values for the healthy male population. The CASA system demonstrated the ability to reduce subjective errors and showed no significant differences in sperm count and motility compared to traditional methods.

**Conclusion:**

This study provides precise sperm morphology reference values that enhance male infertility diagnostics and treatment, particularly in sperm selection for assisted reproductive technologies like ICSI.

## 1 Introduction

According to a new report released today by the World Health Organization: Globally about 17.5 percent (about one in six) of the adult population suffers from infertility (1). Among the many etiologies of male infertility, abnormal sperm quality is considered one of the most common causes; the importance of male reproductive and sperm function is increasingly recognized (2).

Sperm morphology analysis provides an assessment index of sperm health status. It has become an indispensable tool in the assessment of fertility and the diagnosis of male infertility (3). The World Health Organization (WHO) has issued standardized guidelines on sperm morphology analysis and has identified the proportion of morphologically normal sperm as a key indicator for semen analysis. However, the current methods of sperm morphology analysis are based on manual, which is highly subjective, individualized, and inefficient (4, 5).

CASA(Computer-Assisted Sperm Analysis) can rapidly analyze many of sperm samples and significantly reduce the error caused by manual subjectivity (6). These devices are considered useful tools for the rapid analysis of many samples, reducing inter-operator variability and therefore providing a high degree of repeatability (7). However, sperm morphology analysis still faces several challenges in practical applications. Among them, the lack of measurement data of normal morphology spermatozoa is an important factor hindering the use of CASA for sperm morphology analysis (8). Realizing the precise quantification of sperm morphology characteristics can help provide a more comprehensive and detailed assessment of sperm quality and provide a scientific basis for clinical decision-making.

Providing reference values for sperm function and testing for different populations is an important topic and direction for clinical testing (8). Sperm morphology analysis results vary due to different preparation techniques, choice of staining methods, and differences in the evaluation systems. Therefore, laboratories should establish appropriate reference value ranges based on self-population and laboratory methods.

The current reference values for sperm head morphometry are based on standards published by the World Health Organization (WHO) in 2010(5th) and 2021(6th) (3, 9) The 5th and 6th editions of the WHO manual guidelines describe only three sperm head morphology parameters (L, W, and L/W ratio), limiting the description of spermatozoa in various clinical situations.

To address this situation, the present study used a healthy fertile population, according to WHO standards, using the Papanicolaou staining method recommended by the WHO manual, examined by experienced (more than 10 years of relevant experience) morphological examiners, and measuring the relevant parameters of the sperm using the Suiplus analysis system. This will provide a reference value for sperm morphology in a healthy fertile population, and establishing the normal sperm morphology reference range based on strict standards can improve the accuracy of sperm morphology diagnosis by CASA. This will improve the assessment of male fertility and optimize sperm selection.

## 2 Materials and methods

### 2.1 Study participants

This study was conducted with the approval of the Human Subject Ethics Committee of the China Family Planning Science Research Institute (NRIFP2023024). Informed consent was obtained from all subjects. We confirmed that all methods were carried out following relevant guidelines and regulations.

Following the WHO criteria, we evaluated fertility with the time to pregnancy less than 12 months. Therefore, we set the following inclusion and exclusion criteria. Inclusion Criteria: (i)healthy young men of fertile couples whose spouses were pregnant within 12 months; (ii)completion of a physical examination, blood, urine and semen tests and a questionnaire; (iii)examined between 01/2023 and 06/2024. Exclusion Criteria: (i) underlying genetic or other diseases (sexually transmitted diseases, reproductive tract infections, cardiovascular diseases, obesity) that severely affect male fertility; (ii)long-term exposure to radioactive rays or other toxic substances; (iii)taking medications (antibiotics, immunosuppressants, systemic corticosteroids, or chemotherapeutic drugs) in the last 3 months; (iv)semen infections, and urinary tract infections; (v) smoking cigarettes, using drugs, and abusing alcohol.

### 2.2 Computer-assisted sperm analysis and viability measurement

Human sperm samples were obtained by masturbation after 2-7d of abstinence. Samples were allowed to liquefy at room temperature, no longer than 1h. Basic semen analyses were performed according to WHO manual standards, and CASA(SSA-II PLUS, China) was analyzed with reference to the previous method (10).

### 2.3 Papanicolaou staining and sperm morphology analysis

Sperm fixation and staining were performed according to WHO manual standards (11). Samples were fixed by immersion in 95% ethanol (v/v) for at least 15 minutes. The smears were rehydrated stepwise in 80% ethanol (v/v) for 30 seconds, 50% ethanol (v/v) for 30 seconds, and purified water for 30 seconds. Nuclei were stained with Harris’s hematoxylins for 4 minutes, and excess dye was removed with water. To de-stain the cytoplasm, the smears were dipped in acidic ethanol 4–8 times, then rinsed in water to restore the blue color of the nuclei. Scott’s solution was used, followed by washing in cold tap water for 5 minutes. For cytoplasmic staining, smears were dehydrated in 50%, 80%, and 95% ethanol (v/v) sequentially, then stained with G-6 orange for 1 minute. After dehydration in 95% ethanol, the smears were stained with EA-50 green for 1 minute to stain the cytoplasm and nucleoli. Final dehydration was done in 95% ethanol (v/v) and 100% ethanol, followed by clearing in xylene for mounting.

Followed with WHO 6th, the spermatozoa morphology was by the three experienced technicians (Fang Zhou, Longlong Fu, Shi Qiu). At least 1000 spermatozoa per semen sample were counted manually by the operators, and the analysis was performed on two slides per sample to ensure consistency.

### 2.4 Sperm morphometric measurements by SSA-II plus systems

The experimental setup consisted of several key components. A computer system equipped with an Intel i5 11th generation processor (2.4 GHz), and NVIDIA 1660 graphics card. The imaging was conducted using an Olympus CX43 upright microscope (Olympus, Tokyo, Japan) with a 10x eyepiece and a 100x oil immersion objective lens, equipped with a 1x C-type interface. A CMOS-based microscope camera with a 1/1.2-inch sensor, 1920 × 1200 resolution, and a frame rate of over 70 fps was used for capturing images. The platform for slide scanning was the BM8000 automated microscope scanning platform, which supports up to eight standard slides with XYZ-axis automatic movement and focus adjustment capabilities.

The specimen is magnified with a 100x objective lens and digitized by the camera. The SSA-II Plus system processes and analyzes the image. The system calculates the focal plane by capturing a series of Z-axis images ≥ 40 fps, selecting the clearest to identify the optimal focal plane. In single-field sperm analysis, the system locates, counts, and segments sperm, calculating various parameters and classifying them as normal or abnormal. After determining the primary focal plane, the system adjusts the objective up and down over a short distance, capturing and analyzing morphological images for each field. Typically, 400 sperm or 100 fields are analyzed, with customization options. The overall morphology is assessed, and image measurements are validated using a microscope micrometer.

### 2.5 Data collection and statistical analyses

Demographic and anthropometric information (age, height, weight, and body mass index) was collected and assessed.

At least 1000 spermatozoa per semen sample were randomly captured. The length of the sperm head (HL, μm), is defined as the distance between the two furthest points along the long axis of the head, typically in an elliptical shape. The width of the sperm head (HW, μm) is determined by the perpendicular distance between the two furthest points on the short axis, which represents the shortest axis of the head. The area of the sperm head (HA, μm2) is calculated based on the contour of the head, while the perimeter (HP, μm) refers to the length of the boundary surrounding the head. The ellipticity is the ratio of the length to the width of the sperm head (L/W), which reflects its shape. The acrosome area (AcA, μm^2^), measured in square micrometers, refers to the area of the acrosome, the cap-like structure on the sperm head. The acrosome ratio (AcR,%) is the ratio of the acrosome area to the midpiece area, providing insight into the relative sizes of these two regions of the head. The neck length(NL, μm) is the length of the neck segment of the sperm, and the neck width(NW, μm) is the width at the widest part of the neck. The insertion angle (IA,°) is the angle between the symmetry axis of the neck and the long axis of the sperm head.

Descriptive statistics and statistical analysis were performed using SPSS for Windows, version 15.0 (SPSS Inc., Chicago, IL, USA).

## 3 Results

### 3.1 Study participants and semen parameters

A total of 21 fertile males (within less than 12 months of pregnancy waiting time) were included in this study. Semen samples from the study participants were collected for analysis within 1-3 months after successful delivery by their female partner. The mean age of the study participants was 30.29 ± 4.69 year, height 175.52 ± 6.89cm, weight 76.76 ± 8.35, while BMI 22.5 ± 2.5 and the abstinence days were 4.62 ± 1.39 days.

The routine parameters of semen analyzed are detailed in **Table 1**. The data were normally distributed, as assessed by the Shapiro-Wilk test, justifying the use of parametric tests like the t-test. A paired t-test was used to validate the two methods of analysis, and no significant differences were seen in the parameters.

**Table 1:** The semen parameters of fertile male participants.

	Basic semen examination	CASA	
Semen volume (ml)	4.52 ± 1.87		
Sperm concentration (10^6^ per ejaculate)	59.99 ± 22.58	58.05 ± 21.34	*P*=0.1302
Total sperm number (10^6^ per ejaculate)	261.28 ± 138.65	271.09 ± 144.77	*P*=0.0908
Progressive motility (PR,%)	51.52 ± 13.37	49.87 ± 14.50	*P*=0.1501
Total motility (PR+NP,%)	54.71 ± 12.40	59.09 ± 18.11	*P*=0.0554
Normal forms(%)	9.90 ± 4.56	10.06 ± 4.91	*P*=0.6408
Velocity along the curvilinear path (VCL, µm/s)		22.63 ± 15.65	
Velocity along the straight-line path (VSL,µm/s)		8.92 ± 5.14	
Velocity along the average path (VAP, µm/s)		13.65 ± 7.94	
The amplitude of the lateral displacement of the head (ALH, µm)		2.00 ± 1.85	

### 3.2 Sperm morphologic diagnostic results

Three experienced sperm morphology diagnosticians (FZ, LLF, and SQ) annotated the collected sperm images three times, harmonized the diagnosis, and classified the individual sperm morphology. A total of 29,994 spermatozoa were collected from the 21 study participants. Consistency tests were performed by three operators on so of the sperm classification. Where discrepancies existed, they were discussed by the three operators and the final results were given. The number of normal morphology sperm was 2995(9.98%). The specific data of sperm morphology analysis of the 21 study subjects are detailed in **Table 2**.

**Table 2:** The specific data of sperm morphology analysis.

Researcher No.	Total number of sperm counted	Number of normal sperm		Number of normal head sperm		Number of normal midpiece sperm		Number of normal Principal sperm		Number of ERC sperm	
T001	1535	191	12.44%	194	12.64%	1355	88.27%	1464	95.37%	28	1.82%
T002	1509	193	12.79%	208	13.78%	1276	84.56%	1455	96.42%	53	3.51%
T003	1505	167	11.10%	171	11.36%	1368	90.90%	1434	95.28%	56	3.72%
T004	1207	129	10.69%	135	11.18%	1051	87.08%	1010	83.68%	79	6.55%
T005	1519	155	10.20%	166	10.93%	1312	86.37%	1433	94.34%	59	3.88%
T006	1543	247	16.01%	260	16.85%	1387	89.89%	1463	94.82%	31	2.01%
T007	1515	160	10.56%	182	12.01%	1190	78.55%	1442	95.18%	28	1.85%
T008	1073	146	13.61%	152	14.17%	991	92.36%	1026	95.62%	37	3.45%
T009	1534	135	8.80%	142	9.26%	1336	87.09%	1460	95.18%	87	5.67%
T010	1498	106	7.08%	111	7.41%	1314	87.72%	1439	96.06%	46	3.07%
T011	1082	215	19.87%	222	20.52%	993	91.77%	1034	95.56%	37	3.42%
T012	1181	46	3.90%	48	4.06%	1061	89.84%	1134	96.02%	105	8.89%
T013	1519	160	10.53%	171	11.26%	1327	87.36%	1447	95.26%	37	2.44%
T014	1512	41	2.71%	44	2.91%	1335	88.29%	1435	94.91%	45	2.98%
T015	1149	47	4.09%	48	4.18%	1047	91.12%	1104	96.08%	38	3.31%
T016	1506	298	19.79%	312	20.72%	1354	89.91%	1441	95.68%	70	4.65%
T017	1508	79	5.24%	88	5.84%	1368	90.72%	1435	95.16%	45	2.98%
T018	1511	37	2.45%	39	2.58%	1370	90.67%	1445	95.63%	58	3.84%
T019	1540	82	5.32%	89	5.78%	1352	87.79%	1454	94.42%	97	6.30%
T020	1527	190	12.44%	99	6.48%	1406	92.08%	1443	94.50%	114	7.47%
T021	1521	171	11.24%	177	11.64%	1353	88.95%	1449	95.27%	29	1.91%
Ave ± SD	1428.28 ± 168.56	142.61 ± 70.29		145.61 ± 73.37		1264.09 ± 142.56		1355+169.8		56.14 ± 26.29	

### 3.3 Characteristics of sperm head size and shape

The number of normal head morphology sperm were 3059(10.19%), normal midpiece sperm were 26546(88.50%), normal principal were 28447(94.84%), while the number of sperm with Excess residual cytoplasm (ERC) was 1179(3.93%). Detailed data are shown in **Table 3**. In accordance with the WHO manual recommendations, we counted head morphology normal spermatozoa related head morphology (L, W, L/W) and region area data separately. Neck morphology normal spermatozoa data, and tail main segment morphology spermatozoa were counted. And the descriptive analysis of the sperm characteristics of all the normal sperm (**Table 4**).

**Table 3:** Sperm morphological classification and count of subjects.

Researcher No.	Total number of sperm counted	Number of normal sperm		Number of normal head sperm	Head %	Number of normal midpiece sperm	Mid %	Number of normal Principal sperm	Pri %	Number of ERC sperm	
T001	1535	191	12.44%	194	12.64%	1355	88.27%	1464	95.37%	28	1.82%
T002	1509	193	12.79%	208	13.78%	1276	84.56%	1455	96.42%	53	3.51%
T003	1505	167	11.10%	171	11.36%	1368	90.90%	1434	95.28%	56	3.72%
T004	1207	129	10.69%	135	11.18%	1051	87.08%	1010	83.68%	79	6.55%
T005	1519	155	10.20%	166	10.93%	1312	86.37%	1433	94.34%	59	3.88%
T006	1543	247	16.01%	260	16.85%	1387	89.89%	1463	94.82%	31	2.01%
T007	1515	160	10.56%	182	12.01%	1190	78.55%	1442	95.18%	28	1.85%
T008	1073	146	13.61%	152	14.17%	991	92.36%	1026	95.62%	37	3.45%
T009	1534	135	8.80%	142	9.26%	1336	87.09%	1460	95.18%	87	5.67%
T010	1498	106	7.08%	111	7.41%	1314	87.72%	1439	96.06%	46	3.07%
T011	1082	215	19.87%	222	20.52%	993	91.77%	1034	95.56%	37	3.42%
T012	1181	46	3.90%	48	4.06%	1061	89.84%	1134	96.02%	105	8.89%
T013	1519	160	10.53%	171	11.26%	1327	87.36%	1447	95.26%	37	2.44%
T014	1512	41	2.71%	44	2.91%	1335	88.29%	1435	94.91%	45	2.98%
T015	1149	47	4.09%	48	4.18%	1047	91.12%	1104	96.08%	38	3.31%
T016	1506	298	19.79%	312	20.72%	1354	89.91%	1441	95.68%	70	4.65%
T017	1508	79	5.24%	88	5.84%	1368	90.72%	1435	95.16%	45	2.98%
T018	1511	37	2.45%	39	2.58%	1370	90.67%	1445	95.63%	58	3.84%
T019	1540	82	5.32%	89	5.78%	1352	87.79%	1454	94.42%	97	6.30%
T020	1527	190	12.44%	99	6.48%	1406	92.08%	1443	94.50%	114	7.47%
T021	1521	171	11.24%	177	11.64%	1353	88.95%	1449	95.27%	29	1.91%
Sum	29994	2995	9.98%	3058	10.20%	26546	88.50%	28447	94.84%	1179	3.93%
Average	1428.285714	142.619		145.619		1264.095		1355		56.14285714	
standard deviation	168.5651633	70.2983		73.3693		142.5591		169.8		26.29122613	

**Table 4:** Distributions of the morphometry in normal sperm.

Sperm morphologic parameters	Mean ± Sd	Percentile				
		5th	25th	50th	75th	95th
Length(μm)	4.22 ± 0.30	3.72	4.01	4.23	4.44	4.71
Width (μm)	2.74 ± 0.21	2.4	2.59	2.75	2.89	3.09
L/W	1.54 ± 0.10	1.38	1.47	1.54	1.61	1.71
Head area(μm^2^)	9.00 ± 1.17	7.11	8.14	9	9.85	10.94
Ellipticity(%)	0.71 ± 0.13	0.52	0.6	0.7	0.82	0.92
Acrosome area(μm^2^)	4.00 ± 1.10	2.3	3.16	3.92	4.75	5.98
Posterior area (μm^2^)	4.99 ± 0.78	3.66	4.51	5.02	5.53	6.24
Circumference (μm)	9.98 ± 0.67	8.86	9.51	10.01	10.5	11.08
Acrosome ratio(%)	0.44 ± 0.09	0.3	0.38	0.44	0.5	0.59
Mid-section length (μm)	3.57 ± 0.54	3.36	3.36	3.36	3.53	4.18
Mid-section width (μm)	0.87 ± 0.72	0.12	0.3	0.62	1.32	2.62
Insertion angle (°)	11.98 ± 11.26	0.82	3.78	8.12	13.93	39.19

## 4 Discussion

The accuracy and consistency of sperm morphology have been a pressing issue in reproductive male medicine. In recent years, with the rapid development of computerized imaging and artificial intelligence (AI) technologies, significant improvements have been brought to sperm morphology diagnosis (12, 13). Sperm morphology diagnosis still has some limitations in clinical application, such as the subjectivity of testing criteria, diagnostic inconsistencies between different laboratories, and unclear relationship between morphology and sperm function. Laboratory technicians need to undergo appropriate training, testing and control procedures before evaluating sperm morphology for patients, and the long learning curve and workload do not meet clinical needs (14, 15). These deficiencies affect the accuracy and clinical guidance value of morphological analysis. And, the lack of data from healthy fertile populations has limited the development of related technologies to some extent (8, 16).

In this study, we measured normal sperm morphology data of fertile populations based on the Papanicolaou staining technique and the SSA-II Plus diagnostic system.

The staining technique is a crucial step in sperm morphology analysis. By enhancing the contrast of sperm cells, Papanicolaou staining makes abnormal sperm with different morphologies more conspicuous, thus improving the accuracy and consistency of diagnosis (17). The method can effectively highlight the morphology of the sperm head and tail, with strong staining contrast, and can significantly enhance the visibility of the cell structure, so it is widely used in the diagnosis of male infertility (5). It is widely used in the diagnosis of male infertility. It has become a staining method recommended and promoted by the WHO manual. Therefore, Pap staining was selected in this study. The related data provided more favorable data for the future promotion of sperm staining for diagnosis.

CASA is capable of analyzing sperm count and motility, and has gradually become an important technical tool in sperm research and male infertility diagnosis in China by virtue of its accurate, rapid and comprehensive diagnostic capability (18, 19). Our study also shows that SSA as an advanced sperm analysis device, there is no statistically significant difference in the evaluation of sperm count and motility from the methods recommended in the WHO manual. The results of this study show that for total motility, the observed trend (p=0.0554) may become significant with a larger sample size, and we recommend further studies to confirm these findings. However, due to the complexity of sperm identification and classification, CASA still has a lot of room for in-depth research in the diagnosis of sperm morphology (20).

Currently, abnormalities in sperm morphology usually rely only on a subjective assessment of “smooth, regular, and largely oval shape.” (5, 9) The ability of researchers to more accurately measure sperm morphology data, particularly the multiple dimensions of sperm head length, width and ellipticity, allows for a more objective and accurate assessment (7). Measuring the degree of fit of the sperm head to the standard ellipse allowed for a more precise morphometric analysis, avoiding the vague descriptions of the past and improving the diagnostic value of sperm morphology. Several studies (17, 21, 22), have reported sperm head size parameters (length, width and roundness). These studies have established reference values for sperm head size, but they vary across populations. The present study further enhanced the reliability of the sperm samples used and reduced potential bias by ensuring that all participants had conceived naturally within the past year. This study also included data related to the sperm tail to further refine the sperm morphometric data.

This study provides the first reference data on sperm morphology in a healthy male population by combining the Papanicolaou staining method and the SSA-II Plus computer-assisted sperm analysis (CASA) system. This study ensured the fertility of the study population by selecting men who successfully conceived within 12 months as a sample, thus increasing the clinical relevance of the data. And by measuring multidimensional morphological parameters such as length, width, area, circumference, ellipticity, and acrosome area of sperm head. These new parameters provide a more comprehensive perspective for the quantitative analysis of sperm morphology. Establishing these reference values makes sperm morphology analysis more precise, objective and efficient, and further enriches the evaluation criteria of sperm quality. These new data not only provide a more scientific basis for future male infertility assessment but also offer potential clinical value for sperm selection, especially in the application of assisted reproductive technologies such as single sperm injection (ICSI).

However, although this study provides important data and methods for the field of sperm morphology, there are still some limitations. First, the sample size of this study was relatively limited, with all participants coming from research centers in mainland China. Second, this study did not explore the applicability of different sperm morphology classification criteria in different populations. Therefore, future studies should consider expanding the sample size to cover more regions and different age groups of men and to include related populations such as clinically adverse pregnancies. In-depth studies should be conducted to investigate the relationship between sperm morphology measurements and clinical pregnancy outcomes (e.g., pregnancy rates, live birth rates, etc.), particularly through longitudinal studies to track the predictive power of sperm morphology on fertility outcomes. This will ensure that the results are representative of the wider population and provide more accurate data to support the assessment of male fertility on a global scale.

Sperm morphometric data provide a more objective and precise tool for sperm morphology diagnosis, in male infertility assessment and assisted reproductive technology, especially the sperm selection in ICSI. In this study, we measured normal sperm morphology data of fertile populations based on the Papanicolaou staining technique and the SSA-II Plus diagnostic system. These new standards and parameters help to better assess sperm function and fertilization potential, and provide an important basis for the development of individualized treatment plans.

## Data Availability

The original contributions presented in the study are included in the article/supplementary material. Further inquiries can be directed to the corresponding author.

## References

[1] Cox CM Thoma ME Tchangalova N Mburu G Bornstein MJ Johnson CL. Infertility prevalence and the methods of estimation from 1990 to 2021: a systematic review and meta-analysis. Hum Reprod Open. (2022) 2022:hoac051. doi: 10.1093/hropen/hoac051 36483694 PMC9725182

[2] Skakkebaek NE Rajpert-De-Meyts E Buck Louis GM Toppari J Andersson A-M Eisenberg ML. Male reproductive disorders and fertility trends: Influences of environment and genetic susceptibility. Physiol Rev. (2016) 96:55–97. doi: 10.1152/physrev.00017.2015 26582516 PMC4698396

[3] World Health Organization. WHO Laboratory Manual for the Examination and Processing of Human Semen. 6th ed. Geneva: World Health Organization (2021).

[4] Compiling Group of Chinese Experts on Sperm Morphology Analysis from the Reproductive Testing Branch of China Sexology Association. Chinese experts’ consensus on sperm morphology analysis. Chin J Androl. (2023) 37:5–13. doi: 10.3969/j.issn.1008.0848.2023.03.002

[5] Xu YH Lu JC Tang SS. Effects of six kinds of sperm staining methods on human sperm size and evaluation of their staining effects. J Clin Lab Anal. (2022) 36:e24794. doi: 10.1002/jcla.24794 36441612 PMC9757014

[6] Cherouveim P Velmahos C Bormann CL. Artificial intelligence for sperm selection-a systematic review. Fertil Steril. (2023) 120:24–31. doi: 10.1016/j.fertnstert.2023.05.157 37236418

[7] Yüzkat M Ilhan HO Aydin N. Multi-model CNN fusion for sperm morphology analysis. Comput Biol Med. (2021) 137:104790. doi: 10.1016/j.compbiomed.2021.104790 34492520

[8] Jia YL Wu YB Yu L Zheng Y Yang TT Wang YY. Normal sperm head morphometric reference values in fertile Asian males. Asian J Androl. (2024) 26:315–20. doi: 10.4103/aja202356 PMC11156450 38048168

[9] World Health Organization. WHO Laboratory Manual for the Examination and Processing of Human Semen. 5th edn. Geneva: World Health Organization (2010).

[10] Fu L Fang F Guo Y Ma J Wang S Gu Y. Combined analysis of the transcriptome, proteome and metabolome in human cryopreserved sperm. World J Mens Health. (2024) 42:610–9. doi: 10.5534/wjmh.230091 PMC11216965 38164029

[11] Calogero AE Cannarella R Agarwal A Hamoda TA Rambhatla A Saleh R. The renaissance of male infertility management in the golden age of andrology. World J Mens Health. (2023) 41:237–54. doi: 10.5534/wjmh.220213 PMC10042649 36649928

[12] Kim J Lee SJ. Digital in-line holographic microscopy for label-free identification and tracking of biological cells. Mil Med Res. (2024) 11:38. doi: 10.1186/s40779-024-00541-8 38867274 PMC11170804

[13] Lustgarten Guahmich N Borini E Zaninovic N. Improving outcomes of assisted reproductive technologies using artificial intelligence for sperm selection. Fertil Steril. (2023) 120:729–34. doi: 10.1016/j.fertnstert.2023.06.009 37307892

[14] Björndahl L Kirkman Brown J other Editorial Board Members of the WHO Laboratory Manual for the Examination and Processing of Human Semen. The sixth edition of the WHO Laboratory Manual for the Examination and Processing of Human Semen: ensuring quality and standardization in basic examination of human ejaculates. Fertil Steril. (2022) 117:246–51. doi: 10.1016/j.fertnstert.2021.12.012 34986984

[15] Fu LL Liu Y Wang XW Zhou F Guo Y Lu WH. The current situation of semen analysis in China, based on a survey of 296 laboratories. Asian J Androl. (2024) 26:220–1. doi: 10.4103/aja202346 PMC10919429 37800924

[16] Fu L Lou Y Guo Y Zhou F Ma J Wang S. Seminal plasma microbiomes, sperm parameters, and cryopreservation in a healthy fertile population. Front Microbiol. (2024) :1401326. doi: 10.3389/fmicb.2024.1401326 39403085 PMC11471716

[17] Maree L du Plessis SS Menkveld R van der Horst G. Morphometric dimensions of the human sperm head depend on the staining method used. Hum Reprod. (2010) 25:1369–82. doi: 10.1093/humrep/deq075 20400771

[18] Baldi E Gallagher MT Krasnyak S Kirkman-Brown J Editorial Board Members of the WHO Laboratory Manual for the Examination and Processing of Human Semen. Extended semen examinations in the sixth edition of the WHO Laboratory Manual for the Examination and Processing of Human Semen: contributing to the understanding of the function of the male reproductive system. Fertil Steril. (2022) 117:252–7. doi: 10.1016/j.fertnstert.2021.11.034 34986981

[19] Li Y Lu T Wu Z Wang Z Yu T Wang H. Trends in sperm quality by computer-assisted sperm analysis of 49,189 men during 2015-2021 in a fertility center from China. Front Endocrinol (Lausanne). (2023) 14:1194455. doi: 10.3389/fendo.2023.1194455 37529601 PMC10390301

[20] Deshmukh SS Shakya B Chen A Durmus NG Greenhouse B Egan ES. Multiparametric biophysical profiling of red blood cells in malaria infection. Commun Biol. (2021) 4:697. doi: 10.1038/s42003-021-02181-3 34103669 PMC8187722

[21] Katz DF Overstreet JW Samuels SJ Niswander PW Bloom TD Lewis EL. Morphometric analysis of spermatozoa in the assessment of human male fertility. J Androl. (1986) 7:203–10. doi: 10.1002/j.1939-4640.1986.tb00913.x 2427496

[22] van den Hoven L Hendriks JC Verbeet JG Westphal JR Wetzels AM. Status of sperm morphology assessment: an evaluation of methodology and clinical value. Fertil Steril. (2015) 103:53–8. doi: 10.1016/j.fertnstert.2014.09.036 25450299

